# What defines a healthy gut microbiome?

**DOI:** 10.1136/gutjnl-2024-333378

**Published:** 2024-09-25

**Authors:** Matthias Van Hul, Patrice D Cani, Camille Petitfils, Willem M De Vos, Herbert Tilg, Emad M El-Omar

**Affiliations:** 1 Louvain Drug Research Institute (LDRI), Metabolism and Nutrition research group (MNUT), UCLouvain, Université catholique de Louvain, Brussels, Belgium; 2 Walloon Excellence in Life Sciences and BIOtechnology (WELBIO) department, WEL Research Institute, Wavre, Belgium; 3 Institute of Experimental and Clinical Research (IREC), UCLouvain, Université catholique de Louvain, Brussels, Belgium; 4 Human Microbiome Research Program, Faculty of Medicine, University of Helsinki, Helsinki, Finland; 5 Laboratory of Microbiology, Wageningen University, Wageningen, The Netherlands; 6 Department of Internal Medicine I, Gastroenterology, Hepatology, Endocrinology & Metabolism, Medizinische Universitat Innsbruck, Innsbruck, Austria; 7 Microbiome Research Centre, St George and Sutherland Clinical Campuses, University of New South Wales, Sydney, NSW, Australia

**Keywords:** intestinal microbiology, nutrition, barrier function

## Abstract

The understanding that changes in microbiome composition can influence chronic human diseases and the efficiency of therapies has driven efforts to develop microbiota-centred therapies such as first and next generation probiotics, prebiotics and postbiotics, microbiota editing and faecal microbiota transplantation. Central to microbiome research is understanding how disease impacts microbiome composition and vice versa, yet there is a problematic issue with the term ‘dysbiosis’, which broadly links microbial imbalances to various chronic illnesses without precision or definition. Another significant issue in microbiome discussions is defining ‘healthy individuals’ to ascertain what characterises a healthy microbiome. This involves questioning who represents the healthiest segment of our population—whether it is those free from illnesses, athletes at peak performance, individuals living healthily through regular exercise and good nutrition or even elderly adults or centenarians who have been tested by time and achieved remarkable healthy longevity.

This review advocates for delineating ‘what defines a healthy microbiome?’ by considering a broader range of factors related to human health and environmental influences on the microbiota. A healthy microbiome is undoubtedly linked to gut health. Nevertheless, it is very difficult to pinpoint a universally accepted definition of ‘gut health’ due to the complexities of measuring gut functionality besides the microbiota composition. We must take into account individual variabilities, the influence of diet, lifestyle, host and environmental factors. Moreover, the challenge in distinguishing causation from correlation between gut microbiome and overall health is presented.

The review also highlights the resource-heavy nature of comprehensive gut health assessments, which hinders their practicality and broad application. Finally, we call for continued research and a nuanced approach to better understand the intricate and evolving concept of gut health, emphasising the need for more precise and inclusive definitions and methodologies in studying the microbiome.

## Definition of a healthy gut

**Figure 1:**
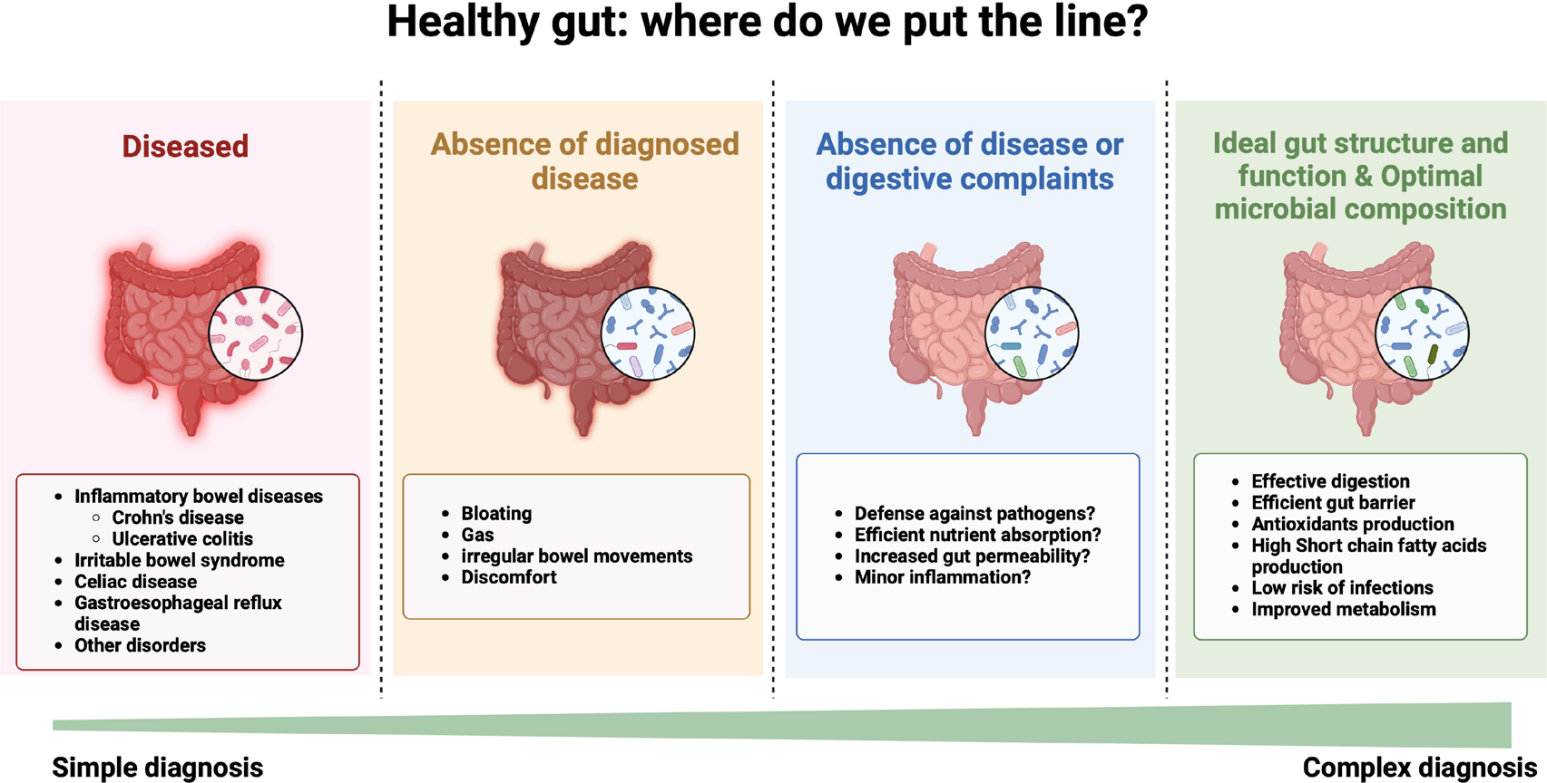
Spectrum of gut health from diseased to optimal gut functionality. The definition of a healthy gut varies depending on scientific/medical perspective. The narrowest definition focuses pragmatically on the ‘absence of a diagnosed disease or disorder’. A broader definition, the ‘absence of disease or digestive complaints’, acknowledges subclinical issues. The broadest definition, ‘a healthy gut has an ideal gut structure and function, including an optimal microbial composition’, encompasses the gut’s impact on the host. Created with BioRender.com.

 The definition of a healthy gut varies and can be somewhat subjective, as it intertwines with both scientific perspectives and individual health experiences. Almost 15 years ago, the concept of 'gut health' was increasingly used in the medical literature and considered as a major interest in preventive medicine.^1^ Some experts define a healthy gut from a functional or clinical viewpoint, considering it as not having any diagnosed digestive diseases or disorders. This viewpoint focuses on the lack of detectable medical conditions affecting the gastrointestinal (GI) tract, such as inflammatory bowel diseases (IBD) like Crohn’s disease and ulcerative colitis, irritable bowel syndrome (IBS), coeliac disease, gastroesophageal reflux disease (GERD), and other functional or structural disorders. This definition is pragmatic and clinically useful because it establishes a clear, although basic, criterion for gut health: if an individual does not suffer from any known GI diseases or disorders, their gut could be considered ‘healthy.’ However, this narrow approach may overlook the fact that not all gut health issues manifest in ways that meet the criteria for a formal diagnosis (figure 1). Moreover, it goes beyond the WHO definition of healthy that is more than the mere absence of disease or infirmity and is defined as a state of complete physical, mental and social well-being (https://www.who.int/about/governance/constitution). Importantly, even within this clinical definition, the role of a balanced and diverse gut microbiota in preventing such diseases cannot be overlooked.

A wider perspective on gut health considers the absence of digestive complaints as a key indicator. Symptoms like bloating, gas, irregular bowel movements and discomfort may not always be linked to a specific disorder but can indicate suboptimal gut health (figure 1). Therefore, a healthy gut, by this definition, is not only ‘*free from diagnosed diseases*’ but also ‘*operates without causing any discomfort or signs of dysfunction’*. This definition also implicitly relies on a well-balanced gut microbiota, as imbalances often lead to these discomforts. However, certain indicators of a compromised gut health, like increased permeability of the gut barrier or minor inflammation, might not immediately manifest through clear symptoms (figure 1). Yet, they are still undesirable as they are associated with elevated health risks and/or could lead to the development of diseases or disorders in the future.

**Figure 2:**
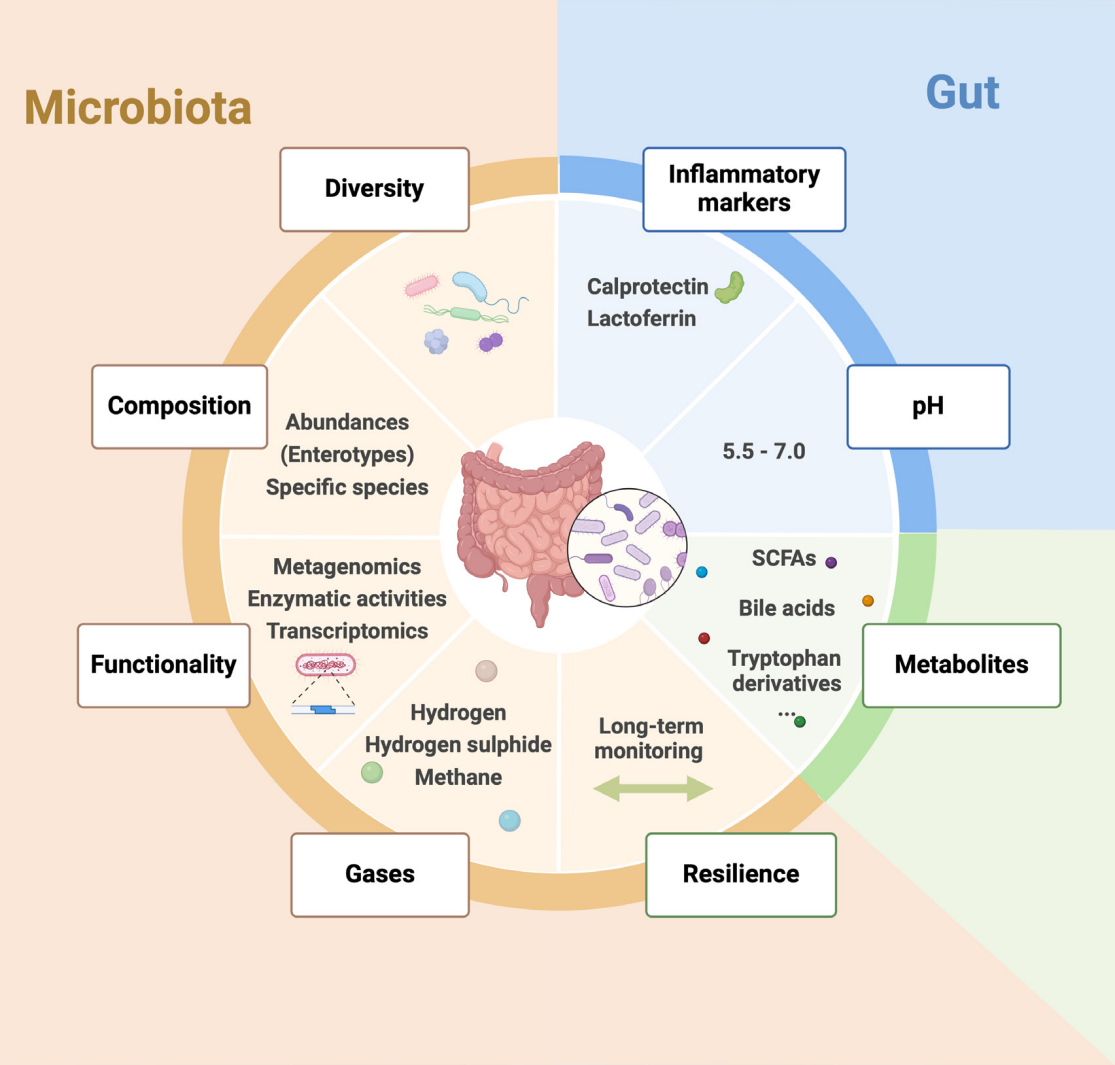
Potential markers of a healthy gut microbiota. A healthy gut microbiota is characterised by a diverse and balanced community of microorganisms that perform vital functions for the host. Several key markers have been proposed to assess these, including microbial diversity, composition (abundances, enterotypes, specific species), functionality (metagenomics, enzymatic activities, transcriptomics) and resilience (through long-term monitoring). Production of important metabolites like SCFAs, bile acids and tryptophan derivatives, as well as gases (hydrogen, hydrogen sulfide, methane), are potential indicators. Additionally, gut health indicators such as pH levels and the presence of inflammation markers (like calprotectin, lactoferrin) can be used for assessing overall gut health. SCFAs, short-chain fatty acids. Created with BioRender.com.

The third and most comprehensive interpretation focuses on the ideal structure and function of the gut, encompassing the composition of the gut microbiome, since the microorganisms in the GI tract play a critical role in digestion, nutrient uptake, energy harvest, vitamin synthesis, inflammatory modulation and host immune status. This holistic view covers the physical health and integrity of the GI tract, that should ensure efficient absorption of nutrients and form a robust defence against harmful substances. It also involves the gut’s functional capabilities, like effective digestion and immune response, and a balanced and diverse microbiome (figure 2). This perspective emphasises that microbiome diversity, the presence of beneficial versus harmful bacteria (opportunistic) and the gut’s role in immune function, mental health and disease prevention are crucial for overall well-being. These broader criteria appreciate the gut’s role in overall well-being and recognise that a person might not have a diagnosed GI disorder but still experience suboptimal gut health due to an imbalance in the microbial structure and function, poor dietary habits, stress or other lifestyle factors. This perspective highlights the critical balance and interplay between the gut’s physical structure, its operational functions, and the complex community of microbes that it harbours.

This comprehensive interpretation of gut health contributes to a multifaceted understanding of gut health, emphasising different aspects of what it means to maintain a well-functioning digestive system. However, the framework required for evaluating and achieving optimal GI health is complex and goes beyond mere clinical assessments and everyday experiences of comfort and well-being (figure 1). To fully understand and define a healthy gut microbiome, it is essential to consider these diverse factors and their interdependencies.

## The first three lines of defence to maintain host health

**Figure 3:**
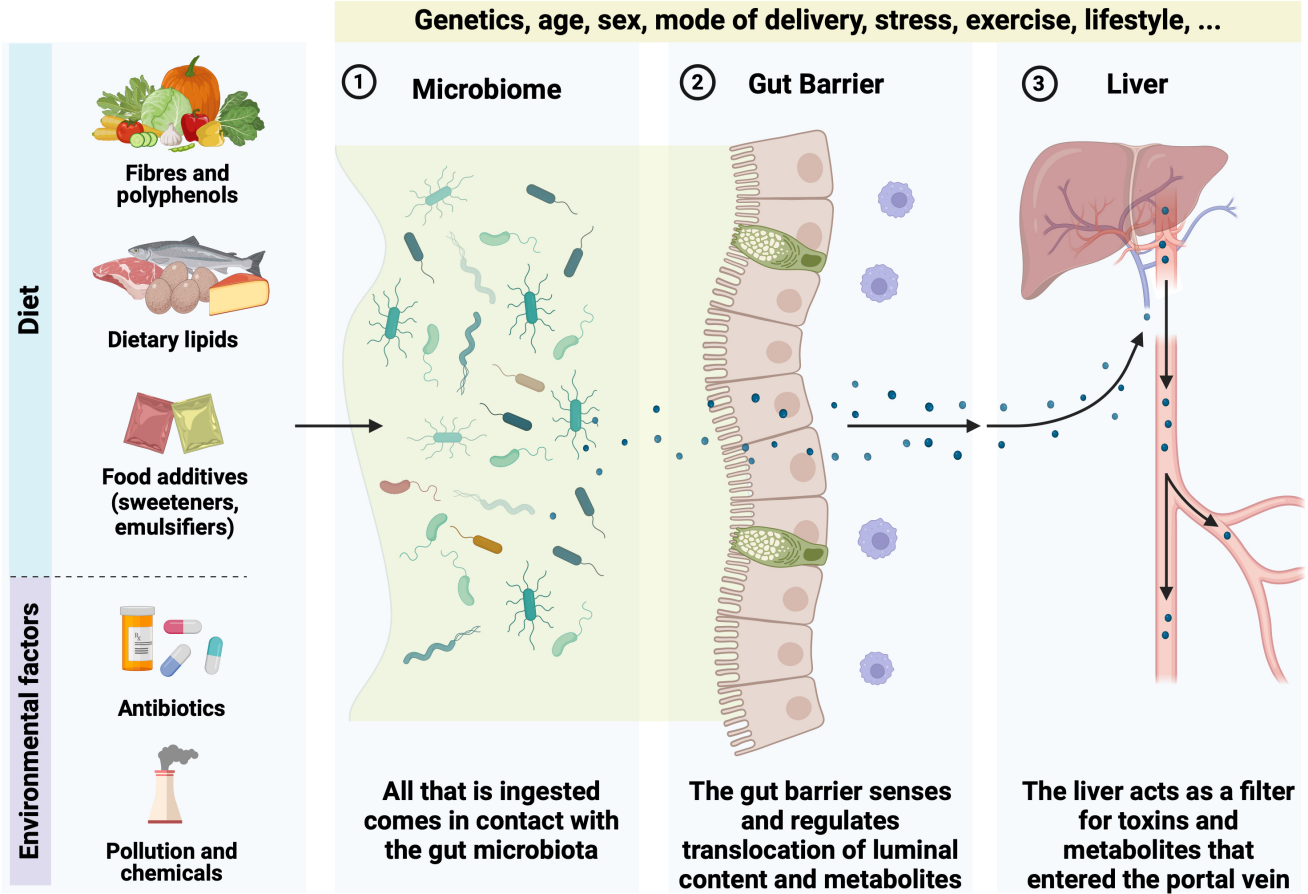
The three ‘lines of defence’. Diet and environmental factors influence the microbiome (1), which interacts with the gut barrier (2) to regulate the translocation of luminal components and metabolites. The liver (3) acts as a filter for toxins and metabolites entering through the portal vein. An unhealthy state, associated with alterations in gut microbiota composition, may lead to increased intestinal permeability (‘leaky gut’), allowing pathogens and toxins into the bloodstream. Genetics, age, sex and lifestyle factors modulate these processes. Created with BioRender.com.

Maintaining health in the face of environmental pressures such as diet, pollutants and toxins is a complex and dynamic process that involves multiple physiological systems (figure 3). Among these, the GI tract and the liver play pivotal roles. The gut microbiota, the gut barrier and the liver represent three critical lines of defence that work synergistically to protect the host from harmful environmental influences.

## The gut microbiome

The human microbiome encompasses a diverse and dynamic collection of microbes, including bacteria, fungi and viruses, their genetic material, and by-products that establish residence in our bodies from birth, transferring vertically through generations. The gut microbiota performs several key functions essential for maintaining host health. It aids in the digestion of complex carbohydrates, synthesises essential vitamins and regulates the immune system. The microbiota also competes with pathogenic microbes for resources and space, thereby preventing their colonisation and potential invasion. Moreover, the gut microbiota produces a variety of bioactive compounds, including short-chain fatty acids (SCFAs), which exert anti-inflammatory effects and contribute to the integrity of the gut barrier. The balance and composition of the gut microbiota can be significantly influenced by diet, antibiotics and other environmental factors, making it a crucial mediator in the host’s defence strategy.

Exposure to specific microbes and the interplay with the environment play crucial roles in shaping the microbiome, highlighting the interaction between external factors and our bodies. While colonisation occurs across all body sites, the gut harbours the largest microbial populations.^2^ In healthy individuals, host factors such as stomach acidity, bile acid production, gut motility and the immune system are classically seen as the main factors influencing the gut microbiome.^3^,^5^ However, numerous other variables affect the microbial colonisation, including chemical parameters like intestinal pH, oxygen levels, biological factors like mucus production, antimicrobial molecules and antibody presence^6^ (figure 2). From birth until approximately the age of 3–4 years, a person develops their primary resident microbiota,^7^ though the gut microbiome may undergo a more prolonged development, resulting in a composition that is as distinctive as a set of fingerprints.^8^ Taxonomically, bacteria are classified according to phyla, classes, orders, families, genera and species. The main phyla are Bacillota (formerly Firmicutes), Bacteroidota (formerly Bacteroidetes), Actinobacteriota, Pseudomonadota (formerly Proteobacteria) and Verrucomicrobiota, with the top two phyla Bacillota and Bacteroidota representing roughly 90% of gut microbiota. However, the exact proportion of each of these phyla varies significantly between healthy subjects and do not constitute a major marker of diseases.^9^ While gaining insight into the composition and dynamics of the human microbiome, particularly within the gut, is crucial for understanding its contribution to health and disease, we suggest emphasising additional critical aspects beyond mere microbiota composition to delineate a healthy gut microbiome.

Over 10 years ago, Lozupone and colleagues highlighted several fundamental questions regarding the diversity of gut microbiota.^9^ These questions either remained unaddressed or have only been partially explored with the progression of more advanced sequencing technologies. These questions included: *What is the extent of diversity within the human microbiota and microbiome (the total gene content within the microbiota) at both the species level and broader taxonomic categories? Among the characteristics of the microbiota, such as specific species or their functions, which are common across many individuals, and which are unique to a single person? And, how effectively can the functions of a microbial community be predicted by understanding the species composition, and we add, and eventually predict the development of human diseases?* (figure 2).

The extent of diversity within the human microbiome is vast and varies significantly between individuals. Studies have shown that the human gut microbiome can contain hundreds to thousands of different bacterial species, with significant variation at higher taxonomic levels as well.^10^ However, despite this diversity, certain core microbial species are present across a large portion of the population, suggesting a common set of functions essential for human health.^9^

The composition of the gut microbiota generally shows resilience to short-term disturbances, quickly reverting to its original state due to its inherent plasticity.^11 12^ However, prolonged exposure to stressors common in modern lifestyles, such as Western dietary habits, food additives, environmental contaminants and frequent antibiotic use, can lead to chronic alterations in the gut microbiota. These persistent changes may favour the proliferation of more virulent microorganisms, potentially resulting in negative health outcomes for the host.

There is growing evidence linking alterations in the microbiome to a growing number of diseases, including IBD, liver diseases, obesity, diabetes and even some neurological dysfunctions. The concept of imbalance in the microbiome (as defined below) has been associated with disease states. However, while correlations can be identified, establishing causation and predicting disease development based on microbiome composition are complex tasks.^13 14^

The composition of gut microbiota is influenced by a complex interplay of both intrinsic (host) and extrinsic (external-environmental) factors, which significantly impact human health. Host-related factors encompass age and delivery mode, liver metabolism capacities, immune system functionality, production of specific bioactive lipids and physiological conditions, where genetic variations contribute to determining microbial diversity and resilience, influencing disease susceptibility and treatment responses. External factors, including dietary habits, hygiene practices, exposure to pollutants and chemicals, psychological stress and lifestyle elements such as physical activity, sleep patterns and social interactions, also play crucial roles in shaping the microbial landscape (figure 3). Diet is a primary determinant and will be discussed more in detail below, though we will not elaborate on the other external influences that have been reviewed elsewhere.

Understanding these complex influences is essential for developing interventions such as personalised nutrition, probiotics, prebiotics and lifestyle modifications to maintain or restore healthy gut microbiota, ultimately enhancing digestive, immune and mental health outcomes. Therefore, we will discuss the role of host factors, including age and delivery, liver metabolism, specific bioactive lipids produced, diet and overall genetics that play significant roles in determining disease risk factors and the resilience of the microbiome.

## Characteristics of a healthy gut microbiota

Understanding what constitutes a healthy gut microbiota has been a major focus of recent research. Below we discuss what could be considered as markers of a healthy gut microbiota, their advantages and limitations, and address the ongoing challenges in objectively determining what a healthy gut microbiota should look like.

### Diversity

High bacterial diversity, characterised by a large number of different species, is generally considered a marker of good gut health. High microbial diversity contributes to robust digestion, nutrient absorption, metabolite production and immune system regulation. It enhances resilience against disturbances such as antibiotic use and infections and promotes quicker recovery towards a balanced state. Conversely, reduced diversity is linked to various diseases, including IBD, obesity, type 2 diabetes, cardiovascular diseases and certain cancers. Healthy diets and exercise are known to increase microbial diversity, and athletes typically exhibit higher diversity levels.^15^

However, although low diversity might be indicative of poorer health, high diversity does not necessarily equate to better health. There are indeed notable exceptions to the rule, for example, high gut microbial diversity has been associated with extended colonic transit time and the systemic circulation of potentially harmful protein degradation products.^16^ Additionally, the beneficial effects of diversity can vary significantly between individuals due to genetic, environmental, and lifestyle differences (figures2  3).

### Composition

Rather than merely focusing on diversity, the current literature predominantly examines the specific composition of gut microbiota in diseased versus healthy states. This approach provides deeper insights into the microbial alterations associated with various health conditions, enabling researchers to identify specific bacterial taxa that may contribute to or protect against disease. By comparing the relative abundances and functional roles of these microbial communities, scientists can uncover potential biomarkers for disease and develop targeted therapeutic strategies to modulate the gut microbiome for improved health outcomes.

For example, the **Firmicutes/Bacteroidetes (F/B) ratio** (now known as Bacillota/Bacteroidota ratio) has garnered significant attention as a potential biomarker for health. Studies have suggested that an increased F/B ratio may be associated with obesity and metabolic disorders, as Firmicutes may be more efficient in energy extraction from food, potentially contributing to weight gain, whereas a higher proportion of Bacteroidetes has been linked to leanness. Additionally, an increased F/B ratio has been observed in GI conditions such as IBD and IBS. However, the F/B ratio’s reliability as a health indicator is limited by individual variability, the microbiome’s complexity and conflicting evidence across studies.^17^,^19^

**Enterotypes** were discovered as robust clusters categorising individuals based on the predominant microbial communities present in their guts.^20^ This classification was used to provide insights into how different microbial profiles might correlate with various health outcomes, offering a framework for personalised nutrition and medical interventions. Enterotypes have shown to be useful for identifying general patterns in gut health and potential predispositions to certain diseases, thus aiding in the development of targeted therapies and dietary modifications. However, this approach has its limitations and should be used with caution. The concept of enterotypes may oversimplify the complex and dynamic nature of the gut microbiome, which is influenced by numerous factors, including diet, environment and genetics.^21^,^23^ Therefore, while enterotypes can provide valuable insights, they should not be the sole metric for assessing gut health, and their use should be complemented with other comprehensive analyses (figure 2).

The relative abundance of certain bacterial genera at lower taxonomic levels could be a more precise indicator of gut health. For instance, a higher prevalence of genera such as *Bifidobacterium* and *Lactobacillus* (renamed recently^24^) is often associated with beneficial gut functions, including improved digestion and enhanced immune response; whereas an over-representation of genera like *Clostridium* or *Escherichia* can be indicative of a microbiota deviation and associated with GI disorders, such as IBS or IBD. However, there are significant limitations and counterexamples that challenge the reliability of these indicators. The gut microbiome is highly individualised, influenced by a multitude of factors including diet, genetics and environmental exposures, making it difficult to establish universal biomarkers. Additionally, some genera that are typically considered beneficial can be harmful in specific contexts or in individuals with certain predispositions. For example, an overgrowth of *Lactobacillus* can contribute to conditions such as small intestinal bacterial overgrowth (SIBO).^25 26^ Furthermore, the presence of potentially pathogenic genera does not always correlate with disease; some individuals can harbour these bacteria without manifesting any adverse health effects. Therefore, while the relative abundance of bacterial genera provides valuable insights, it must be interpreted with caution, considering the complex and multifaceted nature of the gut microbiome.

### Functionality

If compositional diversity alone is insufficient for assessing the health of the microbiota and the host, functional diversity—the range of functions performed by the microbiota—might be more suited to evaluate the continued proper operation of the gut ecosystem. Indeed, a high diversity of species in the gut does not necessarily translate to a wide range of functional capabilities. When comparing composition versus function, studies examining large cohorts have highlighted that while only approximately 45% of bacterial species are similar between two individuals, their microbiota share 82% common metabolic pathways.^9 27 28^ This significant functional redundancy—capacity of different bacterial species, whether closely related or not, to perform the same functions—suggests that different bacterial compositions can still maintain similar metabolic functions, emphasising the importance of assessing functional diversity over mere compositional diversity for a more comprehensive understanding of gut health. Functional markers such as enzymatic activity and gene content can provide more accurate predictions of physiological states than compositional diversity alone. Enzymatic activity involves the breakdown of complex carbohydrates, proteins, and lipids, while functional gene content reflects the microbiota’s metabolic capabilities. On the other hand, functional redundancy within an individual may itself be an advantage by providing a form of resilience, as the loss of one species may be compensated by another with similar functional capabilities (figure 2).

However, assessing and understanding functional diversity require advanced and expensive techniques such as metagenomics and advanced bioinformatics and often only provide predictive information. Additionally, functional profiles can vary significantly between individuals, complicating the establishment of universal markers.

### Metabolites

Products such as SCFAs, bile acids (BAs) and tryptophan metabolites are frequently referred to when assessing gut health and optimal gut microbiota composition. Particularly, SCFAs, including acetate, propionate and butyrate, which are directly produced by gut microbes during the fermentation of dietary fibres, have been extensively studied due to their pivotal roles in maintaining gut barrier integrity, modulating immune responses and serving as an energy source for colonic cells.^2^ In recent years, a health-promoting role for butyrate has emerged as discussed below. BAs, synthesised from cholesterol in the liver and subsequently modified by gut bacteria, are also valuable indicators of microbial influence on host metabolism and gut integrity. Another significant group is tryptophan metabolites, such as indole and kynurenine, which reflect both microbial and host metabolism, thereby providing a comprehensive picture of gut health.^29^

Despite their utility, several challenges exist in the accurate quantification of these metabolites, necessitating specialised analytical techniques such as gas chromatography-mass spectrometry and liquid chromatography-mass spectrometry. Additionally, variability in metabolite production due to diet, individual microbiota differences and the complex interplay between microbial and host metabolism can complicate the interpretation of these markers. While SCFAs are reliable indicators of fibre intake and general gut health, their levels can fluctuate based on dietary patterns and individual microbiota composition. Similarly, BAs enterohepatic circulation and host genetic factors can affect their utility as gut health markers. Tryptophan metabolites, although providing broad insights, are influenced by systemic health conditions and dietary intake, adding another layer of complexity.

Overall, while SCFAs, BAs and tryptophan metabolites can certainly offer valuable insights into gut health and microbiota composition, their use as markers is tempered by the need for precise measurement techniques and the influence of various external and internal factors, making it difficult to link specific metabolite levels to distinct microbiota profiles and health states.

### Strain specificity

Bacterial strain specificity is crucial due to its significant implications for health, disease and therapeutic interventions. This important concept is often disregarded although under the same microbial species different strains can have vastly different functions. Indeed, the functional diversity among strains mediates differences of efficacy observed in the literature and also partially explains either the deleterious or the beneficial or neutral effects of some bacteria. The best known examples include the case of particular strains of *Escherichia coli*, like enteropathogenic *E. coli* (EPEC) and enterohaemorrhagic *E. coli* (EHEC) that are pathogenic and can cause severe GI disease, whereas other strains are benign and part of the normal gut microbiota.^30^ Conversely, *E. coli* Nissle 1917 is considered as a probiotic with anti-inflammatory properties.^31 32^ Similarly, *Helicobacter pylori* strains vary in their virulence; strains with the *cagA* gene are more likely to cause gastric ulcers and cancer.^33^ Also, *Bacteroides fragilis* includes strains that produce polysaccharide A (PSA), which modulates the host immune system, while other strains do not. PSA-positive strains can protect against colitis by promoting regulatory T cell function, highlighting the importance of strain-specific interactions in immune modulation.^34^ Therefore, we urge taking into account the strain specificity when advocating the presence or the lack of effects of some bacteria on a given health situation. It is also for the reason that safety or health claims by regulatory bodies are always related to strains rather than species.

### Gases

The production of various gases, such as hydrogen, methane and hydrogen sulfide, is another significant indicator of gut health and microbial composition.^29^ These gases are by-products of microbial fermentation processes in the gut. Hydrogen and methane are primarily produced by bacterial fermentation of carbohydrates, with methane production being specifically linked to the activity of methanogenic archaea. Hydrogen sulfide, on the other hand, is produced from the metabolism of sulfur-containing amino acids by specific bacteria (figure 2).

The measurement of these gases has been used in clinical settings to diagnose conditions like SIBO and IBS. The rationale for using gas production as a marker is grounded in the fact that specific gas profiles can indicate particular microbial activities and imbalances. For instance, elevated methane levels and methane-producing organisms have been associated with constipation-predominant IBS (IBS-C), while elevated breath levels of hydrogen and hydrogen sulfide as well as a higher relative abundance of hydrogen sulfide-producing bacteria have been found in individuals suffering with diarrhoea-predominant IBS (IBS-D).^35^

The advantages of using gas production as markers include non-invasive breath tests, which are relatively easy to administer and interpret. However, there are limitations, such as the influence of diet, transit time and individual variability in gas production. Furthermore, interpreting these gases can be complex due to the overlapping symptoms of different GI conditions. Additionally, gases like hydrogen sulfide, despite being indicative of certain bacterial populations, can be difficult to measure accurately due to their reactivity and low concentration in breath samples.

### pH levels

In the colon, a pH around 5.5–7 is often associated with a healthy microbiota. An optimal pH environment supports the growth of beneficial bacteria, inhibits pathogenic species, ensures enzymatic functioning, enhances digestive efficiency and nutrient absorption. However, pH levels can fluctuate based on diet, health status and other factors, making it a variable marker. Fermented foods and high-fibre diets promote SCFAs production, beneficially lowering pH in the colon. Certain medications can also influence pH. Therefore, pH alone does not provide a comprehensive picture of gut health and must be considered alongside other markers (figure 2).

### Inflammation markers

Low levels of inflammatory markers such as calprotectin and lactoferrin in the stool are indicative of a healthy gut.^36^ Low inflammation directly reflects the absence of gut inflammation and related disorders. Measuring inflammation markers in stool is a non-invasive method for assessing gut health. However, inflammation markers can be influenced by a wide range of factors, including infections and dietary changes, potentially leading to false positives. Therefore, these markers must be interpreted in the context of other clinical and microbiota data for accurate assessment^36^ (figures2  3).

### Resilience

The ability of the gut microbiota to maintain a stable composition over time and resist disturbances, such as antibiotics or dietary changes, is crucial for gut health. Resilience indicates a robust and adaptable microbiota capable of maintaining homeostasis and supporting overall health. A resilient microbiota can recover quickly from disruptions, reducing the risk of long-term health issues. However, assessing resilience requires long-term monitoring, which can be logistically challenging and costly. Furthermore, the factors contributing to microbiota resilience are complex and not fully understood, complicating the assessment process.

### Conclusion

While there are several markers indicating a healthy gut microbiota, there is still no consensus on what constitutes a healthy gut microbiota. Each marker has its advantages and limitations, and the complex interplay between these factors adds to the challenge of defining gut health objectively. Emerging research continues to refine our understanding of these markers and their interactions, but the quest for a definitive and universally applicable definition of a healthy gut microbiota remains ongoing.

## The gut barrier

The second line of defence is the gut barrier, a highly selective and dynamic interface between the internal environment and the external world. This barrier consists of a mucus layer, epithelial cells, antimicrobial peptides, tight junctions and immune cells. An intact gut barrier facilitates beneficial interactions between the body and resident gut microbes by enabling efficient nutrient absorption, fending off pathogens and modulating appropriate immune responses. A compromised gut barrier, often resulting from gut microbiota disturbances, inflammation or exposure to toxins, can lead to increased intestinal permeability (referred to as a leaky gut), allowing pathogens and toxins to enter the bloodstream and trigger systemic immune responses (figure 3).

Various bacteria, including *Akkermansia muciniphila* that lives in the mucus layer, have been found to improve the gut barrier function by increasing the expression of tight junction proteins and hereby reducing the circulating lipopolysaccharides (LPS) endotoxin level. These will be discussed below.

### Colonocytes

The colonic epithelial cells, also known as colonocytes, primarily rely on SCFAs like butyrate, which are produced by the fermentation of dietary fibres by gut microbiota. Pioneering studies from Baumler’s team demonstrate that butyrate prompts colonocytes to consume oxygen via the β-oxidation pathway, thereby protecting the host from the proliferation of potentially pathogenic bacteria that can lead to IBD.^37^ Colonocytes are constantly exposed to numerous microbial antigens and metabolites but generally maintain a symbiotic relationship with these microbes, avoiding inflammation. This symbiosis is particularly notable with obligate anaerobic bacteria that thrive in low-oxygen conditions (hypoxia) and produce butyrate, a crucial energy source for colonocytes, helping to sustain a healthy gut.^38 39^ Additionally, butyrate reduces immune cell recruitment and proinflammatory signals.^40^ However, potentially pathogenic bacteria, such as those from the *Enterobacteriaceae* family, can colonise the gut and cause a microbiota deviation, especially after antibiotic treatment. In that context, the changes in the microbiota composition are often characterised by the bloom of facultative anaerobes, capable of surviving in both oxygen-rich and oxygen-poor environments. These gut microbes are a contributing factor to IBD.^41^

A healthy gut is characterised by preserving a perfect intestinal equilibrium and thereby promoting the growth of obligate anaerobes over facultative anaerobes. To do so, maintaining hypoxia in the gut lumen is essential to prevent the expansion of facultative anaerobes, such as pathogenic *Escherichia* and *Salmonella*.^41^ Nitrate, produced by the enzyme nitric oxide synthase 2 (NOS2) expressed in colonocytes, serves as an energy source for facultative anaerobes. Therefore, regulating host-derived nitrate and oxygen is crucial for maintaining symbiosis. Recent findings suggest that obligate anaerobes also prevent the expansion of facultative anaerobes by limiting host production of nitrate and oxygen.^42^ From a mechanistic point of view, this can be achieved when colonocytes are consuming oxygen to β-oxidise butyrate. This contributes to luminal hypoxia by reducing oxygen diffusion into the gut lumen and eventually helps to maintain anaerobic conditions in the lumen. Butyrate also binds to peroxisome proliferator-activated receptor gamma (PPARγ), inhibiting NOS2 and reducing nitric oxide (NO) and nitrate production. Immune cells also respond to butyrate through GPR43 and GPR109^40^ (for review see Mann *et al*^43^). Moreover, PPAR-γ activates mitochondrial β-oxidation in macrophages consuming oxygen as well.^44^

In pathological conditions, low butyrate levels in the lumen lead to decreased PPARγ activity, increased glycolysis and reduced oxygen consumption. This results in higher NOS2 expression, more NO production and increased nitrate availability for specific pathogens. Recent studies have indicated the importance of butyrate-producing bacteria in the gut. Microbiota analysis of two cohorts showed butyrate producers to be associated with reducing the risk of hospitalisation due to infectious diseases.^45^ Moreover, this effect may be causal since administration of the butyrate-producer *Anaerobutyricum soehngenii* was found to improve health in patients with type 2 diabetes .^46^

### Goblet cells and mucus

Among the other components of the gut barrier, it has been shown that the mucus layer plays a major role (for review see Paone and Cani^47^). Mucus primarily consists of various components, including 90%–95% water, electrolytes, lipids (1%–2%), proteins and other substances. This secretion is dilute, aqueous and viscoelastic due to the presence of specialised proteins known as mucins. Mucins are a family of large, complex, glycosylated proteins. These mucins, which make up 1%–5% of the mucus, are the key structural and functional elements (for review see Paone and Cani^46^ and Gustafsson and Johansson^48^). There are different types of mucins in the intestinal tract and besides the composition of the mucins, a healthy gut is characterised by an adequate mucus thickness, which is not (or poorly) penetrable by bacteria. The turnover of the intestinal mucus layer encompasses the processes of mucus synthesis, secretion and degradation. This is a finely tuned mechanism that must be carefully regulated and balanced to maintain proper mucus function (for review see Paone and Cani^47^ and Gustafsson and Johansson^48^) (figure 3).

Recent data show that several prebiotics might also contribute to the regulation of the mucus production, composition and degradation. For example, fructo oligosaccharide (FOS) treatment can prevent high-fat diet (HFD)-induced metabolic disorders by stimulating the production of glucagon like peptides 1 and 2 (GLP-1 and GLP-2),^49 50^ but also likely at least in part, by acting on all the processes of the mucus production.^51^ Prebiotic treatments like 2′-fucosyllactose (2′FL), an abundant component of human milk oligosaccharides (HMOs), have been shown to enhance gut barrier integrity by influencing the mucus layer and contributes to protection against obesity in vivo.^52^ In the context of HFD feeding, 2′FL supplementation can counteract obesity and metabolic alterations. It is associated with changes in the intestinal mucus layer, characterised by increased expression of secreted and transmembrane mucins, glycosyltransferases and alterations in mucin glycans composition.^52^ Therefore, HMOs not only contribute to the maturation of the infant gut barrier,^53^ but in high concentrations they enhance intestinal epithelial cell function, reduce inflammation and metabolic disorders likely through gut microbiota–mucus barrier dependent mechanisms.^52^

### Immune cells

Maintaining a healthy gut barrier is essential for a healthy gut microbiome, as the gut microbiome plays a pivotal role in our overall health, affecting everything from immune responses to metabolic processes.^2 54^ Indeed, substantial research has shown that the gut microbiome and the immune system are in constant communication, with gut microbes influencing the activity of our immune defence mechanisms (B cells, T-cells, myeloid cells, innate lymphoid cell (ILC)).^55^ This interaction is a delicate balance, as imbalances in T-cell activity can lead to a range of health issues, including inflammatory and autoimmune diseases.^56^ Disruptions in the gut microbiota and the resulting microbiome impact are hallmark traits of IBD. This condition is marked by a notably low presence of butyrate-producing bacteria, including *Faecalibacterium* spp or *Roseburia hominis* alongside diminished butyrate levels. Some other bacteria such as *Bifidobacterium* spp are participating in trophic chains with acetate–lactate converting butyrogens, such as *Anaerostipes* or *Anaerobutyricum* spp.

Again, in the context of a healthy gut and to maintain gut immune health, the role of butyrate is crucial (for review see Mann *et al*^43^). It regulates the plasticity of type 3 innate lymphoid cells (ILC3) and enhances interleukin-22 (IL-22) production.^57^ It also plays a significant role in modulating adaptive immunity. Butyrate influences the differentiation of B cells and plasma cells, promoting the production of intestinal IgA. During the differentiation of regulatory T (Treg) cells, butyrate supports the expression of FOXP3 and the secretion of IL-10. Moreover, it shifts the balance between T helper 1 (TH1) cells and TH17 cells by upregulating T-bet expression and downregulating RORγt expression.^58^ Butyrate and propionate also contribute to the maturation of human monocyte-derived dendritic cells and butyrate stimulates the production of antimicrobial peptides by macrophages.^59^

## The liver

The liver, as the third line of defence, is a vital organ responsible for detoxification and metabolic regulation. It processes and neutralises a wide array of environmental toxins, including pollutants, drugs and metabolic by-products (figure 3). Hepatocytes, the main functional cells of the liver, contain a variety of enzymes, such as cytochrome P450 oxidases, which catalyse the biotransformation of lipophilic compounds into more water-soluble forms that can be excreted via bile or urine. The liver also plays a central role in regulating systemic inflammation and immune responses. Kupffer cells, the liver-resident macrophages, constantly surveil and clear pathogens and debris from the blood. Furthermore, the liver synthesises numerous proteins, including acute-phase reactants and coagulation factors, which are crucial for responding to infections and injuries. The liver’s ability to regenerate and adapt to various stresses is fundamental to its role in maintaining homeostasis and protecting the host from environmental insults.

### Liver metabolism and the bidirectional gut–liver axis

The liver and gut microbiota interact closely, highlighting the importance of a bidirectional gut–liver axis in maintaining health and the potential for therapeutic interventions targeting this axis in liver and metabolic disorders.^60^ The portal vein drains the blood from most parts of the GI tract and even in a healthy setting, it is estimated that approximately 10% of blood metabolites are derived from the gut.^28 61 62^ Exposure of a healthy liver to (fragments of) commensals has been proposed to contribute to the development and maintenance of liver immunity including various resident leucocyte populations.^63^ Importantly, BAs production and secretion not only affects immune and metabolic processes in the liver but also crucially modulates the composition and functionality of the gut microbiota thereby contributing to human health.^5^

### Bile acids: prototypic metabolic and gut microbiota-modifying players

The liver produces BAs that are essential for the digestion and absorption of fats and liposoluble vitamins in the intestine. BAs also act as signalling molecules and antimicrobial agents that can influence the composition and function of the gut microbiota.^5 64^ Changes in BAs composition affect the growth of certain microbial populations over others, thus shaping the gut microbiome. The topic of BAs biology is becoming more and more complex as there might exist hundreds of different BAs and >2000 different bile salt hydrolases supporting the overall importance of these metabolic/immune mediators in physiology, health and disease.^5^ BAs exert their functions via interference with intracellular nuclear hormone receptors which are ubiquitously expressed in various human tissues. These nuclear receptors include farnesoid X receptor (FXR) and G-protein coupled receptors such as Takeda-G-protein-receptor-5 (TGR5) and are activated by BAs thereby regulating metabolism of glucose and lipids and various innate and adaptive immune processes. Activation of FXR and TGR5 by BAs in the gut affects release of various gut-specific hormones such as peptide YY (PYY) and GLP-1 which are of fundamental importance for regulation of energy and metabolic processes. BAs are conjugated with taurine or glycine within hepatocytes and excreted into the bile duct and when reaching the intestine, especially the colon, metabolised by the gut microbiota towards secondary BAs. There, various intestinal bacteria deconjugate and dehydroxylate the primary BAs cholic acid and chenodeoxycholic acid towards deoxycholic acid and lithocholic acid.^65^ BAs also affect the structure and function of many intestinal bacteria supporting the growth of certain and inhibiting others. They also regulate the synthesis of intestinal antimicrobial peptides and are crucially involved in the regulation of an intact intestinal barrier.^66^ The importance of BAs biology is also supported by the fact that the presence of certain BAs in the intestine correlates with murine and human longevity. The gut microbiota of centenarians is enriched in bacteria such as *Eubacterium limosum* and relatives that protect from trimethylamines and the production of undesired trimethylamine oxide^67 68^ as well as Odoribacteraceae with the ability to produce unique secondary BAs including various isoforms of lithocholic acid such as iso-, 3-oxo-, allo-, 3-oxoallo- and isoallolithocholic acid.^69^ Isoallolithocholic acid shows potent antimicrobial effects against Gram-positive (but not Gram-negative) bacteria such as *Clostridioides difficile* and *Enterococcus faecium* proposing that BAs pathways might contribute to longevity via such mechanisms.^69^

BAs as therapeutics have the potential to improve various liver pathologies including metabolic dysfunction-associated steatotic liver disease (MASLD).^70^ In this recent study,^70^ the secondary BAs, hyodeoxycholic acid, whose levels are depleted in human MASLD, improved MASLD in various preclinical models and negatively regulated intestinal FXR expression. In the intestine hyodeoxycholic acid modulated the gut microbiome and upregulated certain bacteria such as *Parabacteroides distasonis*.^70^ Nie and colleagues recently identified an entirely new microbial BAs, that is, 3-succinylated cholic acid (3-succ CA), which is processed by a β-lactamase derived from *B. uniformis*. This 3-succ CA is a lumen-restricted metabolite with the potential to increase intestinal *A. muciniphila* relative levels thereby improving metabolic dysfunction-associated steatohepatitis (MASH) progression, since this symbiont alleviates liver inflammation symptoms in preclinical models.^71 72^ Importantly humans with progressive MASH exhibited lower intestinal concentrations of 3-succ CA. Interference with nuclear hormone receptors has entered clinical medicine as multiple FXR agonists have been investigated in various liver diseases including immune-mediated disorders such as primary sclerosing cholangitis (PSC), primary biliary cholangitis and MASLD.^73 74^ Besides BAs many other liver-derived factors such as antimicrobial peptides, gall-bladder-derived surfactant protein D or IgA synthesised in the liver exert their functions partly under the control of commensals and are able to regulate gut microbiota homeostasis in health and disease and thereby contribute to a liver–gut–immune-microbiome axis.^75^,^77^

### Liver immune function and systemic inflammation

The liver plays a crucial role in immune regulation and therefore can deeply influence the gut microbiome through the modulation of systemic and local immunity.^78^ For example, the liver can secrete factors that influence gut permeability, inflammation and mucosal immunity, thereby affecting the microbial communities in the gut. The liver also affects the level of systemic inflammation, which in turn can affect the gut microbiome. Chronic liver diseases, such as MASLD are associated with changes in the gut microbiome, potentially due to increased intestinal permeability and the translocation of microbial products into the circulation, which can lead to liver inflammation and alter liver metabolism.^79 80^

Even in healthy conditions, the liver is continuously exposed to bacterial antigens derived from the intestine despite an intact intestinal gut barrier. Yet, a healthy liver does not produce proinflammatory cytokines such as TNF or IL-1 beta (IL-1β). However, the precursor of one IL-1 family member, that is, IL-1 alpha (IL-1α) is expressed constitutively in a healthy liver and is activated in case of major cell death or acute injury.^81^ IL-1 family proinflammatory cytokines such as IL-1α or IL-1β belong to the most potent proinflammatory cytokines in an organism and in a healthy liver, which is continuously exposed towards bacterial antigens, IL-1 receptor antagonist (IL-1Ra) is constitutively expressed likely to dampen potentially evolving inflammation.^82 83^ Also, other mechanisms have been recently proposed which might explain why a healthy liver is protected from inflammation despite continuous antigen exposure.^84^ In this study, the authors showed that in healthy periportal vein zones, immunosuppressive macrophages produce high levels of the anti-inflammatory cytokine IL-10 and express Marco, a scavenger receptor that restrains proinflammatory pathogen-associated molecular patterns (PAMPs) and damage-associated molecular patterns. Importantly the presence of this type of macrophages was dependent on the gut microbiota and especially *Odoribacteraceae* via production of the secondary BAs isoallolithocholic acid, contributed to their presence. The authors furthermore demonstrated that certain liver diseases such as PSC or MASH were associated with a decrease in these immunosuppressive macrophages.^84^ Exposure towards PAMPs such as the LPS endotoxin which is mainly derived from Gram-negative bacteria remains a continuous threat to a human organism and the liver. As soon as a healthy liver fails to clear gut-derived endotoxins (which appear in the portal vein even in health), it might have consequences for the host as presence of systemic endotoxin results in ‘metabolic endotoxaemia’ causing potentially chronic metabolic inflammation as observed in obesity and related disorders.^85 86^ Endotoxin exerts its proinflammatory functions via various mechanisms including activation of toll-like receptor 4 (TLR4) and various strategies besides the above discussed immunosuppressive macrophages might be needed to counteract and control endotoxin exposure. Liver expression of LPS-binding protein or soluble CD14 may constitute one local mechanism to neutralise hepatic endotoxin effects.^87^ It has also been recently demonstrated that intestine-derived high-density cholesterol 3 is able to inactivate endotoxin especially in the portal tract thereby preventing activation of inflammatory cascades in the liver.^88^ As soon as the liver environment changes, and for example hepatic steatosis develops, various immune mechanisms in the liver might be compromised and endotoxin clearance might be impaired. It has been shown in MASLD that not only liver localisation of endotoxin is increased via co-localisation with TLR4 positive liver macrophages but also endotoxin is detectable in the circulation of those patients.^89 90^ The liver is also exposed to other PAMPs such as DNA derived from viruses and bacteria. Therefore, this organ plays a fundamental role in protecting a healthy organism from overwhelming inflammation driven by intestinal-derived products including bacteria, viruses and their metabolites. Such a protective function may require various yet undiscovered strategies. In addition, other gut-derived factors including dietary factors such as SCFAs might directly or indirectly affect liver immunity.^43^

It has been recognised in the past decade that besides endotoxins, commensals and especially bacteria-derived DNA might be present in various extraintestinal tissues such as the liver. Such evidence, however, is largely derived from experimental murine studies. In a recent study, Leinwand and colleagues demonstrated that a liver microbiome is present in healthy mice being enriched by Proteobacteria.^63^ Interestingly, development of hepatic immunity, especially of natural killer T cells, was directed by certain commensals and included chemokine (C-C motif) ligand 5 (CCL5) signalling. It remains also unclear whether the presence of live bacteria is possible in a healthy human liver, or rather just bacterial fragments such as DNA. Moreover, this all relates to low concentrations of bacterial DNA that may suffer from experimental pitfalls.^91^Commensals might exert beneficial effects in the liver as shown in germ-free (GF) mice studies as they may maintain liver immune homeostasis and were preventive in certain models of liver fibrosis.^92^ The role of bacterial components/commensals in extraintestinal tissues, however, remains controversial and needs further studies.^93^

As soon as the intestinal barrier is disrupted as seen in many chronic disorders, and especially in human obesity and related pathologies, the ‘liver microbiome’ picture might change and indeed a bacterial liver signature has been demonstrated in human MASLD.^90^ We have recently shown that patients with liver cirrhosis and associated hepatocellular cancer exhibit bacterial DNA not only in the liver but also in the circulation.^94^ When certain commensals appear in the liver with pathobiont activities they might affect liver health and foster disease.^95^ Translocation of *Enterococcus gallinarum (E. gallinarum*) triggers liver autoimmunity and, disease is improved by antibiotics and importantly this pathobiont was also detectable in human autoimmune liver disease.^95^ Finally, modulation of the gut microbiome might even be beneficial in advanced liver disease even using certain prebiotics such as lactulose.^96^ This prebiotic not only improved mortality in patients with decompensated liver cirrhosis but also decreased the presence of multidrug-resistant bacteria such as vancomycin-resistant *E. faecium*.^96^ The topic of intestinal bacteria and their interaction with the liver in both health and disease is fascinating and might have major implications for a better understanding and management of many liver diseases in the future.

In summary, the liver interaction with the gut microbiome is multifaceted, influencing and being influenced by various metabolic and immune processes. This bidirectional relationship underscores the importance of a healthy gut–liver axis in maintaining overall health and provides insights into potential therapeutic interventions for liver and metabolic disorders.

## Factors influencing the gut microbiome and health

Various factors influence the composition and functionality of this microbial ecosystem. These factors include diet, antibiotics and medications, age, genetics, lifestyle factors, geography and environment, infections and diseases, birth methods and early life and exposure to toxins and pollutants (for review see de Vos *et al*^2^, Cani *et al*^97^ and Korpela and de Vos)^98^ (figure 3).

### Age and mode of delivery

Age and delivery mode play crucial roles in shaping the gut microbiota, which in turn can have profound effects on host health.^98 99^ Recently, consensus has been reached that the newborn is sterile and there is no evidence for a foetal microbiome.^91^ From the moment of birth, the gut microbiome begins a dynamic process of development influenced by a variety of factors, including the mode of delivery—vaginal birth or caesarean section—and subsequent environmental exposures such as diet and antibiotics.^100^ The early gut microbiome of caesarean section delivered infants is unusual and shows reduced relative levels of *Bacteroides* and *Bifidobacterium* spp, while so-called pathobionts are increased compared with vaginally born infants.^101 102^ Since maternal faecal microbiota transplantation but not vaginal seeding of caesarean section born infants can normalise their gut microbiota and reduce the level of pathobionts, it has been proposed that the origin of the newborn’s gut microbiome is the mothers’ gut.^103^ Recently, the contribution of faecal microbes of the father also has been elucidated and the absence of maternal transmission to the caesarean section delivered newborn is explained by limited physical contact as well as effect of antibiotics.^104^ The initial colonisation process is notably influenced by whether an infant is breastfed or formula-fed, with breastfed infants typically showing a microbiota dominated by rapidly growing *Bifidobacterium* spp, which is less diverse but tailored to metabolise human milk oligosaccharides effectively.^105 106^ During pregnancy, maternal immunity and the metabolites produced by microbes play a crucial role. The transfer of microbes during childbirth, along with the transfer of immune factors, microorganisms and their metabolites through breastfeeding, are essential for the initial microbial exposure and immune system development in early life. Progressive introduction of solid foods induces a diversification of the child’s microbiota that will progressively converge towards its adult composition.^107^ The arrival or outgrowth of new species will trigger an immune response called 'weaning reaction'. This is necessary for the establishment of an immune system that is, at the same time, protective and tolerant towards commensals.^108^

Later in life, the gut microbiota shows alterations with ageing that are different between healthy and unhealthy ageing populations. Centenarians, often used as an example of healthy ageing, regardless of their geographical origin, tend to have similar taxonomic and functional modifications of their gut microbiota.^109^ While useful to gain insight on how the gut microbiota composition is modified in different age settings, these results rarely come from longitudinal studies and should be used with caution. These processes have significant implications for the health of humans, shaping our resistance to diseases and overall health from an early age.^110^

### Diet

Dietary components are among the most significant factors influencing the composition and function of the gut microbiota.^111 112^ This, in turn, impacts the intestinal health and thereby the gut barrier integrity, inflammation and numerous metabolic processes such as energy balance, glucose and lipids metabolism.^2 29 113 114^ Given the number of reviews covering this topic, we focused on the main nutrients having shown an impact on gut barrier function and health.

The effects of various dietary components are discussed and include fibres, polyphenols, prebiotics, HMOs, fatty acids, refined sugars, sweeteners, and emulsifiers, on the gut microbiota and associated health outcomes.

### Fibres

Dietary fibres, found in fruits, vegetables, whole grains and legumes, are non-digestible carbohydrates that escape the digestion in the upper part of the GI tract and may serve as substrates for microbial fermentation in the colon.^115^ This fermentation process produces SCFAs, including acetate, propionate and butyrate. These three SCFAs are the most extensively researched microbial metabolites and are crucial in managing host metabolism. They serve as energy sources for intestinal epithelial cells and regulate several physiological functions, such as insulin signalling, lipid metabolism and immune cell differentiation.^3754 116^,^118^ Mechanistically, butyrate plays a vital role in controlling the environment of intestinal stem cells and in the renewal process of epithelial cell precursors. SCFAs activate specific G-protein-coupled receptors present on intestinal L-cells, namely GPR41 and GPR43, which in turn stimulate the secretion of GLP-1 and and PYY, leading to appetite-suppressing effects^119^ (figure 3).

### Prebiotics

Among the dietary fibre’s family, prebiotics are playing a major role. Prebiotics are defined as ‘substrates selectively used by host microorganisms that confer a health benefit’.^120^ Common prebiotics include inulin, FOS, HMOs and galacto-oligosaccharides. These compounds enhance the growth of beneficial bacteria such as Bifidobacteria and Lactobacilli, which, in turn, produce SCFAs and other metabolites beneficial for gut health and systemic functions.^121 122^ A large body of literature has shown that prebiotics can influence gut health via different mechanisms, including the production of SCFAs. By activating GPR43/41 receptors expressed on the L cells, SCFAs not only enhances the secretion of GLP-1 and PYY as stated above, but also the secretion of GLP-2, a gut peptide known to contribute to maintain the gut barrier function by stimulating the proliferation of intestinal epithelial cells, enhancing tight junctions integrity, stimulating the blood flow.^123^ Overall, the interaction between gut microbes and these molecular actors helps reduce intestinal permeability, improve insulin secretion and sensitivity, decrease food intake, lower plasma lipids and prevent hepatic steatosis and metabolic endotoxaemia, all of which are associated with reduced inflammation (figure 3).

### Polyphenols

Polyphenols are complex bioactive compounds abundant in plant-based foods such as fruits, vegetables, tea, coffee and wine.^124 125^ The phenolic compounds are categorised into two primary groups, that is the flavonoids and the non-flavonoids. Flavonoids are further divided into anthocyanins, flavanols, flavanones, flavonols and isoflavones. On the other hand, non-flavonoid compounds consist of phenolic acids, stilbenes and lignans (for review see Rodríguez-Daza *et al*^126^). Most polyphenols are present in food in the form of esters, glycosides or polymers that cannot be absorbed in their native form, therefore, most polyphenols act locally and are not present in the circulation. Polyphenols are mostly recognised for their antioxidant properties within the gut and their probable role in the prevention of various diseases associated with oxidative stress. However, the genome of certain gut microbes contains a variety of enzymes that play a metabolic role in enhancing the bioavailability and bioactivity of unabsorbed polyphenols. Therefore, also impact on the gut microbiota composition. They promote the growth of beneficial bacteria like *Lactiplantibacillus* (formerly *Lactobacillus*, see above)*, Bifidobacterium, Roseburia *and *Faecalibacterium* spp as well as *A. muciniphila* but also contribute to inhibiting pathogenic bacteria.^126^,^129^ The interaction between polyphenols and gut microbiota also leads to the production of bioactive metabolites that contribute to gut barrier function, anti-inflammatory and anticarcinogenic effects.^29 130 131^ Phenolic metabolites produced by gut microbes include phenylpropionic acid (PPA), which has shown anti-inflammatory properties and reinforces the gut barrier via aryl hydrocarbon receptor (AhR)-dependent mechanisms.^132 133^ Hydroxyphenylacetic acid (HPAA) and 4-Hydroxyphenylacetic acid (4-HPAA) are known for their anti-inflammatory and antioxidant properties, potentially also protecting against certain cancers, cardiovascular disease and obesity prevention.^132 133^ A large variety of other phenolic acid compounds are produced by the gut microbiota from dietary polyphenols, including caffeic acid, ferulic acid and gallic acid that have diverse potential health-protective effects.^131 132^

### Fatty acids

The type of dietary fat consumed significantly influences gut microbiota composition and function. Saturated fatty acids (SFAs), commonly found in animal fats and processed foods, have been associated with a reduction in microbial diversity and an increase in proinflammatory bacteria.^85 86^ Conversely, omega-3 polyunsaturated fatty acids (PUFAs), found in fish oil and flaxseeds, promote the growth of bacteria such as Bifidobacteria and *A. muciniphila*, that have been mostly associated with improved health.^134 135^ PUFAs can be metabolised by bacteria from the Bifidobacteria*, Enterobacter, Lactobacillus* and *Clostridium* genera into hydroxy and keto derivatives. Among them, CLA and HYA have demonstrated beneficial effects in murine models of colitis, obesity and cancer by activating PPARy, PPARa, GPR120 and GPR140 and by regulating peristalsis through activation of EP3.^136^,^139^

Both dietary cholesterol intake and its plasmatic levels are associated with gut microbiota composition.^140 141^ Cholesterol is only partially absorbed in the upper intestines and every day, 1–2 g of cholesterol enters the colon, where bacteria that possess cholesterol-degrading enzymes will convert it to coprostanol and, to a lesser extent, coprostanone.^142^ Although isolating and culturing bacteria that metabolise cholesterol has been challenging, *Dysosmobacter* and other *Oscillibacter* genera were recently identified. These bacteria negatively correlate with humans’ stool cholesterol levels and were shown to metabolise cholesterol to coprostanone in vitro.^143^

### Sweeteners

Non-caloric artificial sweeteners (NASs) are frequently used in these products to enhance their taste and stability. Despite being approved by regulatory bodies, some artificial sweeteners pose health risks. Research indicates that saccharin, sucralose and aspartame can cause glucose intolerance more significantly than glucose itself, with saccharin having the most pronounced impact.^144^ Most NASs pass through the human GI tract without being digested and their encounter with the intestinal microbiota has been linked to changes in the gut microbiota composition and function^144^ (for review see Gauthier *et al*^145^). Metagenomic analysis highlighted an upregulation of pathways involved in LPS biosynthesis, suggesting a potential mechanism by which sweeteners might increase susceptibility to type 2 diabetes.^144^ The same study also found a positive correlation between sweetener consumption and metabolic indicators such as haemoglobin A1c (HbA1c) and blood glucose levels in humans. High intake of refined sugars and artificial sweeteners can adversely affect gut microbiota composition, leading to gut microbiota deviation,^146^ reduce microbial diversity and impair glucose metabolism, potentially increasing the risk of metabolic disorders like diabetes^146^ (for review see Hosseini *et al*^146^ and Ruiz-Ojeda *et al*).^147^

### Emulsifiers

Emulsifiers, commonly used in processed foods to improve texture and shelf life, can negatively impact gut microbiota and gut barrier function. Despite their widespread use, concerns about the safety of emulsifiers have emerged. Specifically, carboxymethylcellulose (CMC) and polysorbate 80 (P80) have been extensively researched for their potential to induce metabolic disorders. Studies have shown that emulsifiers like CMC and P80 disrupt the mucus layer of the gut, leading to increased gut permeability (leaky gut). CMC disrupts the microbial environment by fostering bacterial overgrowth in mice, while P80 facilitates the translocation of *E. coli*.^148^ Pioneering research from Gewirtz and Chassaing revealed that exposure to CMC and P80 predisposed mice to low-grade inflammation and metabolic syndrome and increased their susceptibility to colitis in IL10−/− mice through a mechanism involving the alteration of the mucus layer.^149^ This groundbreaking study demonstrated that specific emulsifiers alter the permeability of the mucus, allowing bacteria to come into closer contact with intestinal epithelial cells. They found that the gut microbiota plays a crucial role, as transferring microbiota from emulsifier-treated mice to GF mice replicated the gut barrier alterations, including changes in the mucus layer, disruption of tight junction proteins and induced metabolic endotoxaemia.^149^ Moreover, in the absence of microbiota, mice were protected from emulsifier-induced gut barrier dysfunction, low-grade inflammation and subsequent metabolic disorders. These findings were further supported by ex vivo studies using a human gut microbiota simulator (M-SHIME), which showed that CMC and P80 exposure increased the proinflammatory potential by raising bioactive flagellin levels through mechanisms involving the gut microbiota.^150^ GF mice receiving this altered microbiota exhibited the same metabolic disorders, confirming the role of the gut microbiota in mediating the adverse health effects of emulsifiers. More recently, in mice they found that daily oral administration of *A. muciniphila* mitigated the phenotypic effects associated with the intake of CMC and P80. These effects included excessive eating, weight gain and dysglycaemia. Furthermore, the administration of *A. muciniphila* also abolished the low-grade intestinal inflammation induced by CMC and P80 consumption.^151^ In proof of concept double-blind controlled-feeding study, Chassaing *et al* investigate in healthy adults, the impact of an emulsifier-free diet compared with the same diet enriched with 15 g of CMC per day for a duration of 11 days.^152^ Compared with the control group, individuals who consumed CMC experienced a slight increase in abdominal discomfort, a deviation of their gut microbiota composition (ie, reduced diversity) and changes in their faecal metabolome, notably a decrease in SCFAs and free amino acids.^152^ Finally, based on data from a large prospective cohort of French adults they identified a clear link between the probability of developing type 2 diabetes and the consumption of various food additive emulsifiers commonly present in processed foods.^153^

### Specific bioactive lipids

Bioactive lipids play a significant role in health and diseases, influencing various bodily functions and regulating inflammation, gut barrier integrity, the enteric nervous system, immune responses and metabolic health.^154^,^159^

These lipids, including PUFAs, endocannabinoids and related congeners, as well as various families of lipids recently identified, can influence various bodily functions and have been linked to the regulation of inflammation, gut barrier, enteric nervous system, immune response and metabolic health.^31 160 161^

The gut microbiota interacts with lipids derived from the host endogenous secretions and desquamation, dietary fats and bacterial lipids.^162^ How host lipids modify the composition and metabolism of gut bacteria is only beginning to be deciphered. Dietary fats impact on the gut microbiota is being investigated and has been discussed in a dedicated paragraph in this review. Genetic modification of the host lipid production also impacts the gut microbiota as shown in models of tissue-specific depletion of NAPE-PLD, a key enzyme to produce endocannabinoids.^163 164^ As the host-gut microbiota is a bidirectional dialogue, gut microbes reciprocally play an important role in maintaining host lipid homeostasis.

Gut microbiota depletion in GF mice illustrates this importance well. GF mice, despite eating more than conventional mice, present an overall lower body fat content, restored on colonisation, in both males and females.^165^ The gut microbiota’s impact on host lipid composition is not limited to fat storage as GF mice exhibit a modification of the quantity and/or ratio of various lipids. Triglycerides, phospholipids, sphingolipids, glycerophospholipids, plasmalogens, endocannabinoids, PUFAs and their derivatives were found to be modified in the adipose tissue, plasma, serum, liver, faeces and intestines.^136166^,^171^ Moreover, in conventional mice, supplementation with beneficial bacteria like *A. muciniphila and Dysosmobacter welbionis* has shown improvements of the gut barrier function, reduced metabolic endotoxaemia and body weight, decreased metabolic inflammation and improved glucose tolerance concomitantly to changes in the host bioactive lipids.^161 172 173^

The gut microbiota mechanisms that influence host lipid homeostasis are still under investigation. These may include modulation of gene expression and epigenetics, and the incorporation of bacterial lipids into host tissues.^174^,^176^ The signalling role of bacterial lipids has gained more attraction, mainly with the identification of the numerous effects of SCFAs and secondary BAs.^65 174^ Recently, the variety of bacterial lipids with bioactive properties was extended to, for example, commendamide, an endocannabinoid-like molecule produced by *Bacteroides* spp, that has immunomodulatory properties,^177 178^ *N*-oeloyl-serinol a GPR119 agonist that stimulates the production of GLP1 and PYY,^179^ GABA-lipopeptides, produced by *E. coli* Nissle 1917 and *Ligilactobacillus murinus,* which are associated with reduced visceral pain in patients with IBS and have analgesic capacities,^160 180^ and C18-3OH, a PPARy agonist that has anti-inflammatory properties and that is produced by *E. coli* Nissle 1917 and *Holdemanella biformis.*^31^ Membrane lipids, mostly studied for their structural importance, participate in gut microbiota–host communication, particularly in immunology. Specific phospholipids and sphingolipids identified in the membrane of *A. muciniphila* and *Bifidobacterium fragilis* have demonstrated immunomodulatory effects in vitro and in vivo.^175181^,^184^

As this field is in its early stage, the necessary fat input for a healthy gut microbiota, the optimal level of bacterial lipids beneficial to the host or how widespread their production is across species remain to be determined. The diverse enzymatic capabilities of the gut bacteria allow the production of structurally diverse lipids. Unlike mammalian lipids, bacterial lipids can be odd-chained (frequently 15, 17 or 19 carbons) and display a broader range of (de)saturation and of polar heads groups.^181 185 186^ This opens the possibilities for new discoveries and a better understanding of the lipid-mediated two-way communication.

In conclusion, the host’s dietary choices profoundly influence the composition and function of the gut microbiota, with significant implications for overall health. Dietary fibres, polyphenols, prebiotics and HMOs generally promote a healthy microbiota and beneficial health outcomes. In contrast, saturated fats, artificial sweeteners and emulsifiers can disrupt microbial balance, impair gut barrier function.

## Conclusion and perspectives

**Figure 4:**
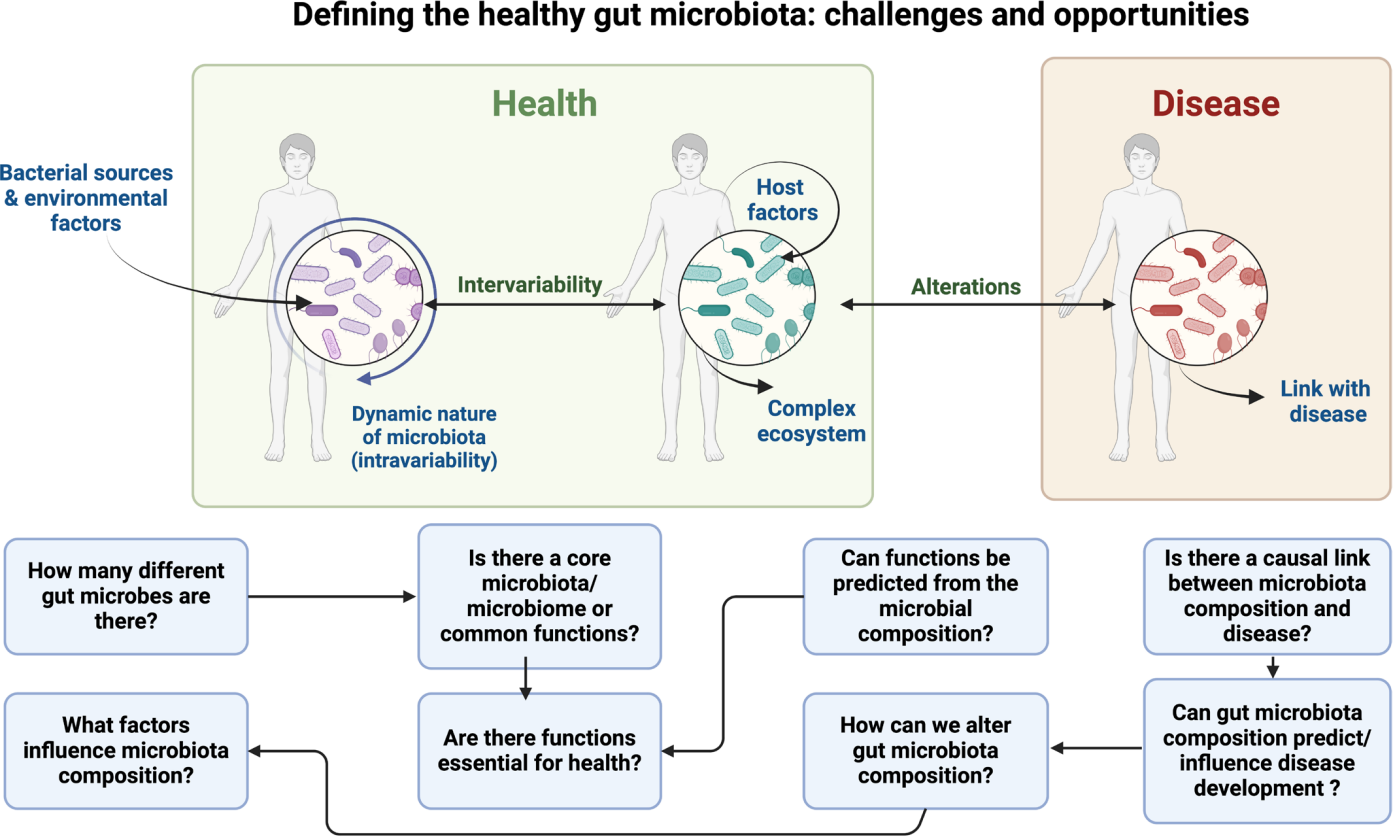
Challenges in defining a healthy gut microbiota. In health, the gut microbiota is influenced by bacterial sources, environmental and host factors, creating a complex and dynamic ecosystem with intraindividual and interindividual variability. Alterations in this balance can link microbiota to disease. Key research questions address the diversity, influencing factors, essential functions and the potential for microbiota composition to predict or influence disease development. Created with BioRender.com.

Despite significant advancements in gut microbiome research, defining the healthy gut microbiota remains a formidable challenge (figure 4). The substantial individual variability, influenced by various factor such as genetics, diet, environment and lifestyle, complicates the establishment of a universal standard for a healthy microbiome. Furthermore, the dynamic nature of the gut microbiome, which evolves over time in response to various factors, underscores the difficulty in capturing a single representative snapshot of health.

The gut microbiome’s immense diversity adds another layer of complexity. Understanding the intricate interactions within this ecosystem and their collective impact on human health is paramount. Current research suggests that the functional capabilities of the microbiome may be more indicative of health than its specific composition, highlighting the need for more precise and affordable methods to identify and measure these functions.

The scarcity of longitudinal data further hinders our understanding, as long-term studies are essential to elucidate the temporal dynamics of the gut microbiome and their implications for health. Additionally, the bidirectional relationship between the gut microbiota and the host’s immune system, metabolism and overall health presents a multifaceted challenge that requires comprehensive investigation.

Environmental and lifestyle factors, such as diet, stress, physical activity and medication use, must be meticulously accounted for when defining a healthy microbiome. These variables, coupled with ethical and practical issues surrounding large-scale data collection and analysis, necessitate standardised methodologies and robust ethical frameworks.

Addressing these challenges will require a multidisciplinary approach, integrating microbiology, genomics, bioinformatics, clinical research and personalised medicine. Future research should focus on developing advanced analytical tools, fostering large-scale longitudinal studies and exploring the functional aspects of the microbiome. Collaborative efforts across scientific disciplines and the incorporation of diverse population data will be crucial in accurately defining and promoting a healthy gut microbiome.

Only by overcoming these challenges can we pave the way for innovative therapeutic strategies and personalised interventions that leverage the gut microbiome to enhance human health and well-being.
